# Possibilities of the Utilization of Ferritic Nitrocarburizing on Case-Hardening Steels

**DOI:** 10.3390/ma14133714

**Published:** 2021-07-02

**Authors:** Jiri Prochazka, Zdenek Pokorny, Jozef Jasenak, Jozef Majerik, Vlastimil Neumann

**Affiliations:** 1 Department of Mechanical Engineering, Faculty of Military Technology, University of Defence, 662 10 Brno, Czech Republic; jiri.prochazka@unob.cz; 2 Department of Manufacturing Technologies and Materials, Faculty of Special Technology, Alexander Dubcek University of Trencin, 911 06 Trencin, Slovakia; jozef.jasenak@tnuni.sk (J.J.); jozef.majerik@tnuni.sk (J.M.); 3 Department of Combat and Special Vehicles, Faculty of Military Technology, University of Defence, 662 10 Brno, Czech Republic; vlastimil.neumann@unob.cz

**Keywords:** chemical heat treatment, ferritic nitrocarburizing, friction coefficient, wear resistance, microhardness profile, depth of diffusion layer, white layer thickness, case-hardening steel

## Abstract

This paper is devoted to the possibilities of the utilization of chosen chemical heat treatment technologies on steels used for manufacturing highly stressed components of military vehicles and weapons systems. The technologies chosen for this research are plasma ferritic nitrocarburizing and ferritic nitrocarburizing in a gaseous atmosphere. These technologies were applied on a steel equivalent 1.5752 (i.e., CSN 41 6426), which is suitable for carburizing. Chemical composition of the steel was verified by optical emission spectrometry. An observation of a microstructure and an assessment of the parameters of obtained white layers were performed by optical microscopy. Morphology and porosity of the surface were observed by electron microscopy. The depth of diffusion layers was evaluated in accordance with ISO 18203:2016(E) from the results of microhardness measurements. A friction coefficient was obtained as a result of measurements in accordance with a linearly reciprocating ball-on-flat sliding wear method. Wear resistance was assessed by employing the scratch test method and a profilometry. The profilometry was also utilized for surface roughness assessment. It was proved that both tested chemical heat treatment technologies are suitable for surface treatment of the selected steel. Both technologies, ferritic nitrocarburizing in plasma and a gaseous atmosphere, are beneficial for the improvement of surface properties and could lead to a suppression of geometrical deformation in comparison with frequently utilized carburizing. Moreover, the paper presents a procedure that creates a white layer-less ferritic nitrocarburized surface by utilizing an appropriate modification of chemical heat treatment parameters, thus subsequent machining is no longer required.

## 1. Introduction

The goal of the paper is to clarify the possibilities of modifying the surface properties of highly stressed components of military vehicles and weapons systems. In the case of these components, including gears, camshafts, pins of crankshafts, connecting rods, parts of weapon systems, etc., high resistivity of the surface against abrasion and corrosion is required. These components are also exposed to combined mechanical stress. Due to these facts, the high hardness and corrosion resistance of the surface is required contrary to the core, which must stay tough enough to provide banding and impact resistance [1]. The requirement of mutually exclusive properties like hardness and toughness is a motivation for utilizing surface heat and chemical heat treatments, coating, or bimetal cladding, which allows for a combination of mentioned properties simultaneously [2,3,4]. The product of these technologies is a surface with properties which differ from an original structure [5]. Surface heat treatments and chemical heat treatments are utilized most often in manufacturing of the mentioned exposed parts. These technologies include surface hardening, carburizing, carbonitriding, nitriding, ferritic nitrocarburizing, boriding, etc. [6,7,8]. Despite the many disadvantages of carburizing, this technology is utilized very frequently. Carburizing is based on a carbon diffusion from the atmosphere, composed of a solid (charcoal powder), liquid, or gaseous medium saturated by carbon, into the surface of a steel. A gaseous atmosphere is used the most often. The disadvantage of carburizing is a high temperature between 800–1000 °C, with the need for subsequent hardening of carburized products [9]. Cracks and geometrical deformations may occur due to the severe decrease in temperature when hardening. The low accuracy of product geometry after carburizing, required for subsequent grinding, is a motivation to find an alternative technology [10]. This paper is devoted to the possibilities of utilizing one such substitutional technology under consideration, namely ferritic nitrocarburizing [11,12]. Contrary to carburizing, the main advantage of ferritic nitrocarburizing is the lower temperature of exposition, generally between 537–600 °C [5]. In the field of thermochemical diffusion techniques, only nitrocarburizing, as well as nitriding, does not require quenching as a subsequent procedure. It results in higher dimension precision without the need for subsequent machining.

## 2. Materials and Methods

One type of steel, carefully selected by an analysis of materials used in the manufacturing of mentioned parts, was investigated by chemical composition verification, microstructure and surface morphology observation, microhardness measurement, friction coefficient measurement and wear resistance assessment.

### 2.1. Materials

According to an analysis of materials used for manufacturing highly stressed components of military vehicles and weapons systems, the steel equivalent 1.5752 (i.e., CSN 41 6426), which is utilized for the manufacturing of mechanical gears, crankshafts, connecting rods, etc., is suitable for carburizing and was therefore selected.

### 2.2. Chemical Composition

The conformity of the chemical composition of the steel was verified by using the advanced CCD optical emission spectrometer Tasman Q4 (Bruker, Billerica, MA, USA), utilizing the Fe110 method. Results are obtained as an average value of five measurements.

### 2.3. Specimen Preparation

Four disk-shaped specimens were cut off from the steel rod in its normalized state. Heat treatment was performed subsequently. Heat-treated steel was determined as an initial state for further chemical heat treatment processes. All specimens were heat-treated in accordance with the parameters shown in Table 1.

Datasheets of the selected case-hardening steel primarily contain parameters of a heat treatment which follows carburizing. Thus, in this case where the carburizing was not performed, the heat-treatment parameters appropriate for commonly utilized low-carbon alloyed steels were chosen in accordance with the literature and experiences of the department.

The heat treatment was followed by grinding and finished by using sandpaper F-1000 according to FEPA. Three of the specimens prepared in this manner were subsequently chemical heat treated.

The first specimen, later used as a reference, was left in a heat-treated state. The second specimen was ferritic nitrocarburized by employing a gaseous atmosphere in a NITREX appliance. The ferritic nitrocarburizing chamber was tempered to 530 °C and the exposure time was set to 6 h. The third specimen was plasma ferritic nitrocarburized. The temperature and process duration were the same as in the previous case.

A cross section of each specimen was cut off by a metallographic saw and molded into thermoplastic powder. Preparation of specimens’ surfaces was completed by grinding and polishing. Polishing was performed by using a velvet and diamond paste with grains of size 0.5 µm.

After a performance of all experimental measurements described in the following text, based on the results, the fourth specimen obtained by plasma ferritic nitrocarburizing, with the same atmospheric parameters but a shortened process duration, was treated and subsequently subjected to the experimental measurements.

### 2.4. Microstructure

First, a microstructure of the specimen in its heat-treated state was observed by using an Olympus DSX500i optical microscope (Olympus, Tokyo, Japan). Due to the reason of migration of carbon and nitrogen into the specimen´s surface, and due to the creation of a specific surface layer during the ferritic nitrocarburizing process, an area influenced by chemical heat treatment was also observed when using the microscope.

A nitride layer composed of three sublayers is formed during ferritic nitrocarburizing. The creation of such a layer is also typical for nitriding. On the top of the surface, the first sublayer, called the compound or white layer, composed of nitrides and carbonitrides of iron, is mostly created. The second sublayer, a diffusion layer, is formed by a dispersion of carbides, nitrides, and carbonitrides of iron and alloying elements. The last sublayer is known as a transition area, which is situated between the diffusion layer and the core microstructure [13,14,15].

The process of ferritic nitrocarburizing is always accompanied by the formation of the white layer as a part of the nitride layer. After supersaturation of this sublayer by carbon and nitrogen, defined interstitial elements diffuse further into the surface [13]. Although the white layer provides protection against corrosion and wear, and improves initial sliding properties after assembly, in some conditions it can negatively affect surface properties [9]. Porosity of the white layer is the most unfavorable factor. Pores decrease corrosion resistance and primarily increase the brittleness of the white layer created on the top of the surface. It may cause the initiation of cracks due to mechanical stress [16]. The porosity of the surface of the chemically heat treated specimens was observed by the TESCAN MIRA 4th generation scanning electron microscope, with a magnification of 10,000×.

### 2.5. Microhardness Measurement

The depth of the diffusion layer was measured in agreement with ISO 18203:2016(E) [17]. NHD (nitriding hardness depth) was evaluated by using microhardness profiles obtained as the result of microhardness measurements conducted by automated microhardness tester LM247 AT LECO (Leco Corporation, St. Joseph, MI, USA). Microhardness profiles were measured on cross sections of specimens. The pattern of impressions was 0.8 mm long. The first impression was performed at a distance of 20 µm from the surface. The step of following impressions was set to 10 µm. The load of the Vickers indenter was 100 g. The resulting microhardness profiles were established from mean values of three measurements of the microhardness profiles, which were performed on each specimen. The depth of the diffusion layer was determined as distance from the surface, where the microhardness of the material´s core increased by 50 HV was measured [17]. Microhardness of the core was determined as a mean value from three impressions, measured at a sufficient distance from a surface, rounded to the nearest multiple of 10 HV [17].

### 2.6. Surface Roughness Measurement

Roughness is deviation from the ideal shape of the surface. Besides machining processes, a chemical heat treatment also plays a role in modifying the surface microgeometry [18]. During application of selected chemical heat treatments, the surface is subjected to high-temperature-enhanced diffusion of elements already present in the material, as well as elements gathered from the surrounding atmosphere which condensate on the surface. In plasma applications, sputtering also affects the surface microgeometry [18]. Due to that fact, surface roughness is also involved in producing values of the friction coefficient. The measurement of roughness profile parameters, i.e., Ra, Rq, Rt, Rz and RSm performed on the profilometer Talysurf CLI1000 (Taylor Hobson Ltd, Leicester, England) by utilizing the contact method was also included in the experimental method. According to ISO 4288, the first measurement, expecting the roughest profile, was performed to obtain values of the Ra and Rz parameters. Those were subsequently utilized for the selection of lr, roughness sampling length (also known as cut-off), and ln, roughness evaluation length, as parameters for further measurements [19]. Values of the roughness profile parameters were finally determined as average values from ten profiles of each measurement.

### 2.7. Measurement of Coefficient of Friction

The friction coefficient, defined as a ratio of friction force and a normal force by which an indenter is loaded, is a parameter which describes the efficiency of a contact movement [12]. Thus, the friction coefficient measurement performed according to the ball-on-flat method, described by the standard ASTM G133-05, was incorporated into the experimental method [20]. The method utilizes ongoing monitoring of parameters, such as normal and friction forces, friction coefficient, acoustic emission, etc., during the measurement, while the indenter moves linearly reciprocally along the specimen surface where the tribological wear track is formed. The Bruker UMT-3 TriboLab instrument (Bruker, Billerica, MA, USA), corresponding with conditions defined by the standard ASTM G133-05, was utilized for the measurement.

The main parameter of such a performed measurement is the friction coefficient, which is plotted to the graph dependent on time. Due to an uneven reciprocating motion of the indenter, obtained data have a square waves profile and hence average values are obtained by subsequent software filtering [21]. Generally, it is possible to distinguish running-in and steady-state wear from the graphs. During the running-in phase the contact surfaces adapt and polish each other [22]. An uneven friction coefficient value is recorded during this phase. When the value stabilize itself, the steady-state wear phase is reached. This period typically takes a long time and in most cases the measurement ends in this phase.

### 2.8. Wear Resistance Assessment

A lower friction coefficient does not always mean better wear resistance. The opposite has already been described, and ferritic nitrocarburized as well as nitrided surfaces are not an exception [23]. For this reason, subsequent measurements were performed on the same instrument by utilizing the scratch test method described in the ASTM G171-03 and ASTM G1624-05 standards [24,25]. The method utilizes Rockwell´s HRC indenter instead of ball-on-flat which was used in the previous case. During each measurement, solely one linear scratch of a predetermined length is performed on the specimen surface. Two modifications of the load of the indenter, such as constant and linearly increasing load, are allowed by the standards. The first measurement was performed with a linearly increasing load in a range from 0 N to 50 N. During the measurement, at a certain distance from an initial point of the measurement some failures of the surface occur. According to the ASTM G1624-05, the first ruptures which occur in the scratch are marked as location L_C1_, which characterize a normal force causing cohesive failures in the white layer. A location where the failures lead to spalling of the white layer is marked L_C2_. According to the result of the first measurement, three values (15 N, 20 N and 35 N) were selected for further measurements performed by an indenter with constant load.

## 3. Results

The beneficial influence of selected ferritic nitrocarburizing on surface properties of steel equivalent 1.5752 (i.e., CSN 41 6426) was experimentally assessed according to chemical composition, microstructure, microhardness, thickness and porousness of the white layer, kinetic friction coefficient, surface roughness and wear resistance.

### 3.1. Chemical Composition

Results of the measurement of chemical composition and the limits of the content of chemical elements mentioned in a datasheet of the steel are listed in Table 2.

The results of the measurement concur with the values listed in the material datasheet of steel equivalent 1.5752 (i.e., CSN 41 6426). Chemical composition has an influence not only on properties of the core of the material but also on the properties of surface layers obtained by chemical heat treatment. According to the literature [9,26], knowledge of the content of elements allows us to predict properties of the resulting layers.

In the case of steel equivalent 1.5752 (i.e., CSN 41 6426), a low content of carbon predetermines the steel for chemical heat treatment, such as carburizing and ferritic nitrocarburizing. By these processes, an increase in the content of interstitial elements, like carbon and nitrogen, in a surface layer is supported. Higher content of carbon and nitrogen in cooperation with alloying elements such as chromium, vanadium and molybdenum leads to the precipitation of particularly hard carbides and nitrides during the diffusion process in the surface layer [26]. Therefore, it can be assumed that the content of chromium in steels may cause an increase in the microhardness of the surface layer after nitrocarburizing. From the measurement, it follows that the steel contains a relatively high amount of nickel. This substitutional element negatively influences the creation of carbides and nitrides as well as the diffusion of carbon and nitrogen into the material [26]. The depth of the nitride layer also depends on the concentration of nitrogen [27]. By utilizing the concentration of nickel, about 3.2 wt.%, it is possible to predict the decrease in the depth of the nitride layer.

### 3.2. Microstructure

**Figure 1:**
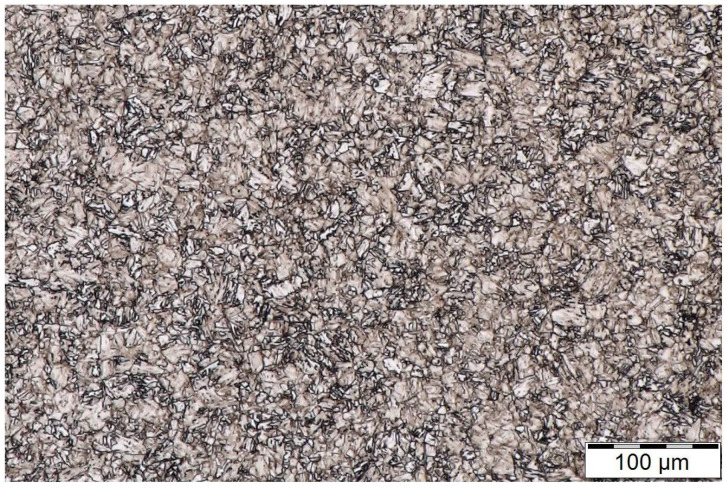
Microstructure of heat-treated specimen. Magnification, 500×.

The microstructure of heat-treated steel, etched by 2% NITAL, which was observed on a cross-sectional specimen by an Olympus DSX500i, is shown in Figure 1. The observed microstructure corresponds with the parameters of the heat treatment applied to the specimens.

**Figure 2:**
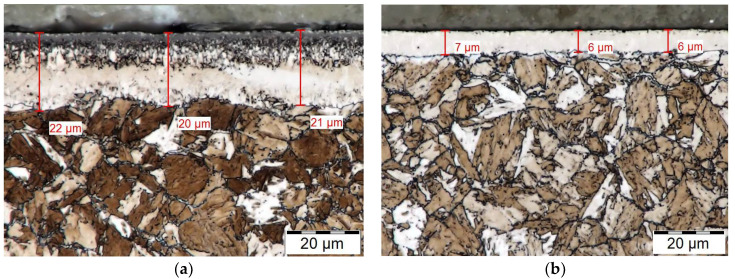
White layers of chemically heat treated specimens. (**a**) Six hours of gaseous ferritic nitrocarburizing and (**b**) 6 h of plasma ferritic nitrocarburizing. Magnification, 2000×.

**Figure 3:**
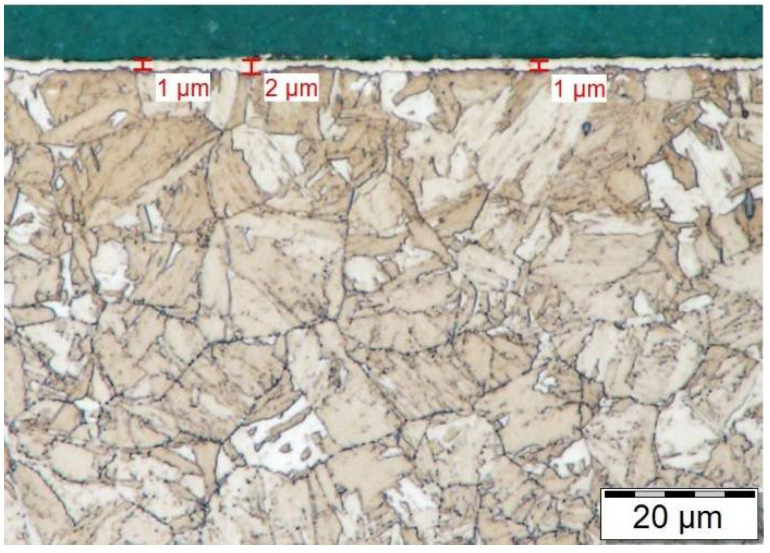
White layer after 4 h of gaseous ferritic nitrocarburizing. Magnification, 2000×.

Chemically heat treated areas obtained by ferritic nitrocarburizing which were observed on the cross-sections of the specimens are shown in Figure 2 and Figure 3. On the left side of Figure 2, the white layer obtained by 6 h of gaseous ferritic nitrocarburizing is shown. The thickness of the white layer oscillates around 21 µm. The porosity of the white layer extends to approximately half of its thickness. The porous part of the white layer is predominantly formed by condensation of particles from the gaseous atmosphere [28]. Due to its properties, mentioned in Section 2.4, it is suitable to remove this porous part of the white layer. This recommendation is unique and dependent on the manner of utilization.

The white layer of the specimen after plasma ferritic nitrocarburizing is shown on the right side of Figure 2. The white layer obtained by plasma ferritic nitrocarburizing is about three times thinner and almost pore-less in comparison with the previous case. Although properties of the white layer obtained after 6 h are better in this case, the paper aims to achieve a white layer-less surface to reduce the possible need of subsequent machining. Therefore, the process of plasma ferritic nitrocarburizing, applied subsequently to the fourth specimen, was shortened to a period of 4 h. An obtained white layer of this nature is visible in Figure 3.

**Figure 4:**
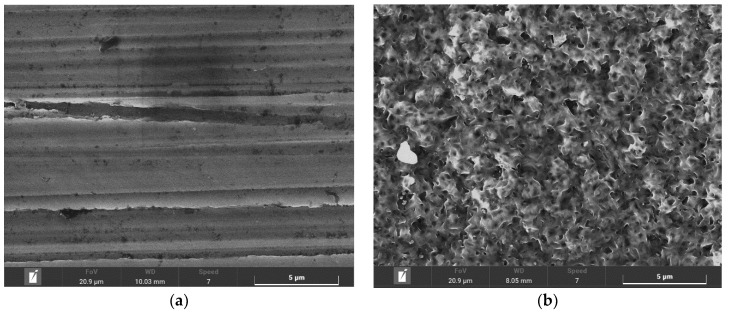

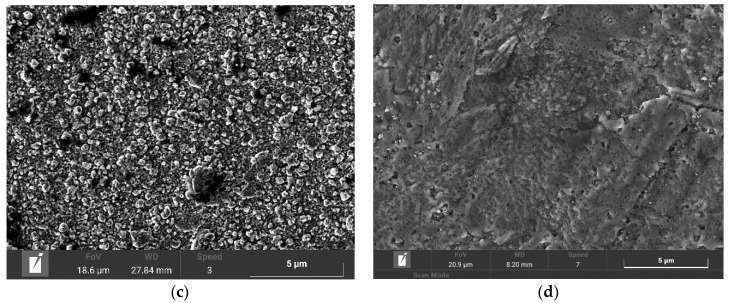
Surface morphology of specimens. (**a**) Heat-treated; (**b**) after 6 h of gaseous FNC; (**c**) after 6 h of plasma FNC; and (**d**) after 4 h of plasma FNC. Magnification, 10,000×.

As is shown in the Figure 3, by reducing the process duration a significant narrowing of the white-layer thickness was achieved. Thus, subsequent machining of the surface is no longer necessary. The statements of the previous paragraphs are further supported by pictures of the surface’s morphology shown in Figure 4, obtained by a TESCAN MIRA 4th generation scanning electron microscope with a magnification of 10,000×.

The surface morphology of the plasma ferritic nitrocarburized specimens is more homogenous, with lower porosity in comparison with those nitrocarburized in the gaseous atmosphere. This finding is in accordance with statements published in [28].

### 3.3. Microhardness

**Figure 5:**
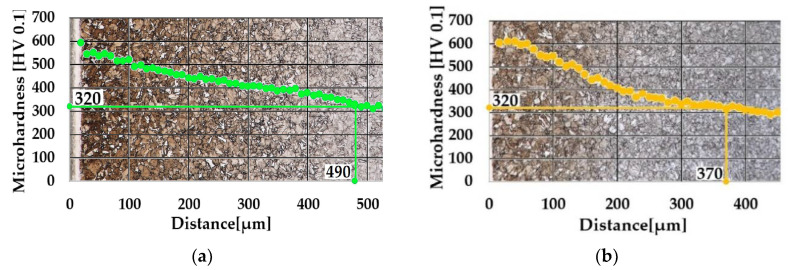
Profiles of microhardness. (**a**) Six hours of gaseous ferritic nitrocarburizing and (**b**) 6 h of plasma ferritic nitrocarburizing. Magnification of microstructure, 500×.

**Figure 6:**
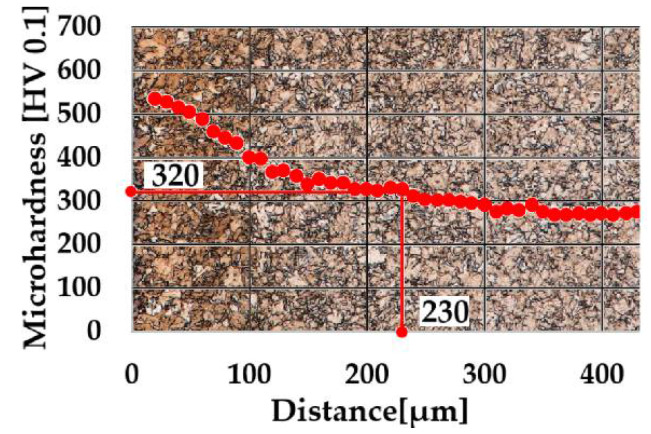
Microhardness profile of layer obtained by 4 h of plasma ferritic nitrocarburizing. Magnification of microstructure, 500×.

As was mentioned in Section 2.5, the measurement of microhardness was utilized for an evaluation of the depth of the diffusion layers. Microhardness profiles composed with appropriate microstructures in the background are shown in Figure 5 and Figure 6.

Microhardness of the core as 270 HV 0.1 was determined; therefore, the limit value of microhardness was equal to 320 HV. As is shown in Figure 5, the depth of the surface layer, obtained by 6 h of gaseous ferritic nitrocarburizing, was determined as NHD 320 HV 0.1 = 490 µm. In the case of 6 h of plasma ferritic nitrocarburizing, the depth of the layer achieved NHD 320 HV 0.1 = 370 µm. While differences in maximum values of microhardness are barely visible, a significant decrease of the NHD in cases of plasma ferritic nitrocarburizing in comparison with the process utilizing gaseous atmosphere was found. This phenomenon could be attributed to the different character of both processes [11]. As is visible in Figure 6, the phenomenon is enhanced with a shortening of the process duration.

Whereas the maximum values of microhardness were preserved in range of 550–600 HV in all cases of chosen chemical heat treatment processes, 4-hour plasma ferritic nitrocarburizing provided lower case depth, i.e., NHD 320 HV 0.1 = 230 µm in comparison with 6-h variants.

### 3.4. Coefficient of Friction and Surface Roughness Measurement

**Figure 7:**
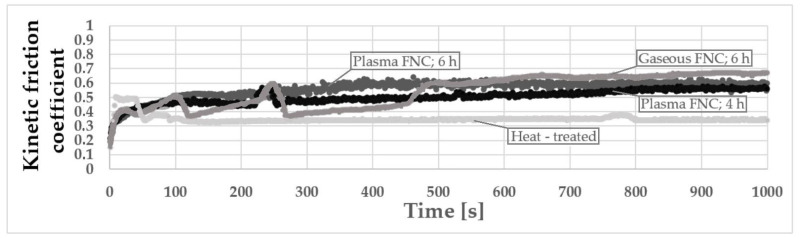
Friction coefficient comparison of heat-treated and ferritic nitrocarburized surfaces.

The friction coefficient measurement was performed subsequently. The measurement parameters were set as follows: normal force = 10 N, duration = 1000 s, frequency = 5 Hz, stroke length = 10 mm, temperature = 23 °C, diameter of tungsten carbide ball indenter = 6.35 mm and dry friction. The results of all measurements are plotted together into the graph listed in Figure 7.

The Figure 7 implies that application of all selected chemical heat treatment processes caused a significant deterioration of surface friction coefficient in comparison with a solely heat-treated surface. To have a better chance of describing the phenomenon, the friction coefficient measurement was accompanied by the measurement of the roughness profile parameters listed in Table 3.

By combining the information mentioned previously, it is possible to state that an increase in the surface microhardness made the surfaces slide by the indenter less easily due to a decrease in the surface formability and also the presence of hard C- and N-based debris in the wear track. The values of the friction coefficient in steady-state wear phase are sorted by order of the values of roughness profile parameters. Thus, the dependance of friction coefficient on surface roughness was confirmed. In the case of the friction coefficient of the gaseous ferritic nitrocarburized surface, the unevenness lasting for a period of almost 500 s could be caused by a warping of the porous part of the white layer during the measurement.

### 3.5. Wear Resistance Assessment

**Figure 8:**
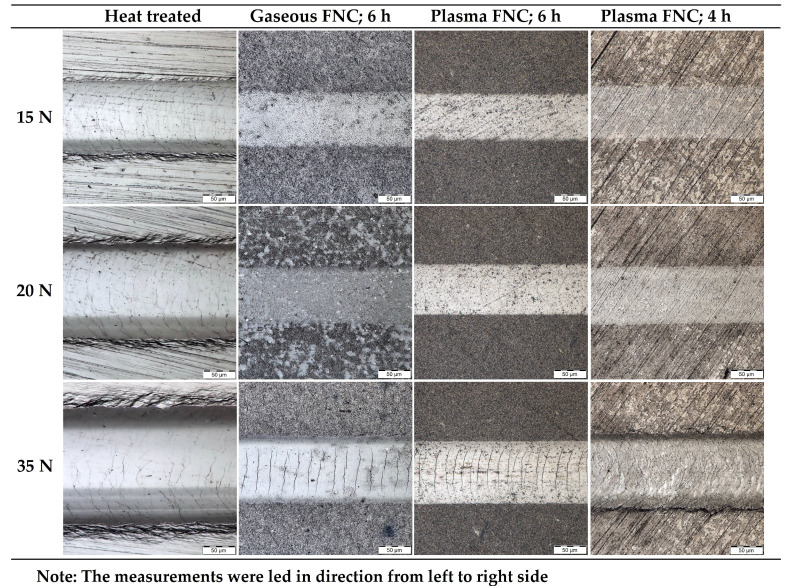
Images of the scratch test wear tracks. Magnification: 1000×.

All specimens were subjected to three scratch test measurements at three different constant loads, i.e., 15 N, 20 N and 35 N. Images of such obtained tracks taken by an Olympus DSX500i opto-digital microscope are visible in Figure 8.

In the case of the heat-treated specimen, similar cohesive failures occurred independently of the load. It could be caused by a higher plasticity of a softer surface in comparison with chemically heat-treated specimens. Due to a lower resistance of the heat-treated surface to being penetrated by the indenter, with increased load much wider tracks were obtained.

In both cases of plasma ferritic nitrocarburizing minor cohesive failures under load 20 N as well as at 35 N were visible. Although, in the case of shortened plasma ferritic nitrocarburizing an observable increase of track width occurred, but in neither case did spalling of the white layer appear.

**Figure 9:**
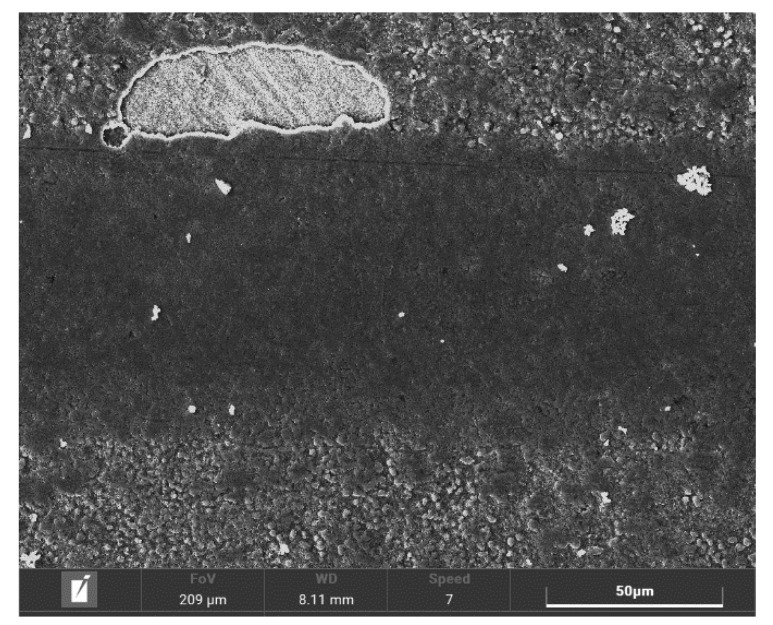
Spalling of the white layer; gaseous ferritic nitrocarburized surface; and load = 15 N. Magnification, 1000×.

Whereas significant cohesive failures were only visible at load 35 N in the case of gaseous ferritic nitrocarburizing, unexpected spalling of the white layer in an area of the track´s edge already occurred at 15 N. The phenomenon was documented by scanning electron microscopy and is visible in Figure 9.

From each scratch wear track, three cross-sectional profiles by a Talysurf CLI1000 profilometer were subsequently taken utilizing the contact method. Parameters of the cross-sectional profiles, such as track width, maximum depth and area of the profile were collected in Table 4 and subsequently utilized for the assessment of surface wear resistance.

The results listed above imply that the application of the selected chemical heat treatments caused a significant decrease in track dimensions. Based on this fact, it could be stated that surface wear resistance was influenced beneficially by the application of the mentioned treatments. From this point of view, the best results were reached in the case of 6-hour plasma ferritic nitrocarburization. The impact of the shortened plasma process on the wear resistance seems to also be positive, and in many cases comparable values of scratch dimensions with ferritic nitrocarburizing performed in a gaseous atmosphere were measured.

## 4. Discussion

The possibility of utilizing selected ferritic nitrocarburizing technologies as a substitutional technology for chemical-heat treatment of case-hardening steel equivalent 1.5752 (i.e., CSN 41 6426), instead of carburizing, was tested. The low content of carbon (0.12 wt.%) predetermine the steel not only for carburizing but also for all surface treatments based on saturation of the surface microstructure by interstitial elements soluble in the ferritic or austenitic lattice. Such chemical heat treatments also include low-temperature treatments, such as nitriding or ferritic nitrocarburizing, which was tested in this paper.

Both technologies, ferritic nitrocarburizing in gaseous atmosphere and ferritic nitrocarburizing in plasma, were applied on the material in a heat-treated state. At first, a 6-hour process duration was selected for both technologies. In both cases, the microstructure in an area near the surface was affected and the layer composed of the white and diffusion layer was observed. Different characteristics of the technologies also caused significant differences in the nitrocarburized layers, especially in the white layers. Whereas in the case of plasma ferritic nitrocarburizing a pore-less white layer was observed with a 6 µm thickness, in the case of gaseous ferritic nitrocarburizing pores appeared in the outer half of its 20 µm thick white layer. The observed differences influenced the choice of a treatment technology of shortened duration for application on the last specimen. Four-hour plasma ferritic nitrocarburizing, subsequently selected to obtain a white layer-less surface, led to the creation of a solely 1 µm thick white layer.

The surface morphology was subsequently assessed by scanning electron microscopy. In the case of gaseous ferritic nitrocarburizing, a highly porous surface was observed. Existence of the porosity in the surface is a consequence of condensation of particles from the gaseous atmosphere, and thus is a consequence of the treatment technology. Therefore, the finding resulting from the observation of the white layer cross section was supported. In the case of plasma-utilizing technology the surface seemed to be smooth, almost pore-less and less rough in comparison with the ground surface of the solely heat-treated specimen. This phenomenon, that was even more visible in the case of shortened plasma ferritic nitrocarburizing, is possible to attribute to the character of the process. During plasma ferritic nitrocarburizing, ionized particles in the nitrocarburizing atmosphere accelerate and bombard the surface. Some surface particles are ejected from the surface under the bombardment, smoothening the surface during the process. 

The microhardness measurements showed that a significant increase of the microhardness with maximum values in a range from 550 HV 0.1 to 600 HV 0.1 by all selected ferritic nitrocarburizing technologies was achieved. It could be attributed to an ability of chromium, also contained in the steel as 0.76 wt.% of it, to create hard and stable nitrides and carbides during the chemical heat treatment processes. By utilizing the microhardness profiles the nitriding hardness depth was determined. The deepest case of NHD, 320 HV 0.1 = 490 µm, was obtained in the gaseous atmosphere. Although in cases of the plasma technology the maximum values of the microhardness were similar, exponentially decreasing microhardness when approaching the core caused a narrowing of the diffusion layer in comparison with ferritic nitrocarburizing in the gaseous atmosphere, where the microhardness decreased linearly. Hence, the ferritic nitrocarburizing potential of the gaseous atmosphere seems to be a little greater in comparison with the selected plasma ferritic nitrocarburizing. The lowest NHD 320 HV 0.1 = 230 µm was found in the case of the shortened process of plasma ferritic nitrocarburizing. Thus, the assumption relating to the interstitial element diffusion retardation, caused by the great amount of nickel (3.2 wt.%) contained in the steel was confirmed, and in connection with the short process duration led to the creation of the shallow nitrocarburized layer.

In all cases of ferritic nitrocarburizing, a significant increase of the friction coefficient was visible. The graph of the kinetic friction coefficient reflects an increase in the surface microhardness as well as the surface roughness, which both contribute to the surface friction coefficient modification. The lowest values of the roughness profile parameters were measured on the shortened plasma ferritic nitrocarburized surface. The friction coefficient measured on the same surface, the lowest of the chemically heat-treated surfaces, could be affected solely by the low roughness of the surface. Contrarily, the highest friction coefficient appeared in the case of gaseous ferritic nitrocarburizing, where the highest values of roughness profile parameters were also found. It also complies with the observed surface morphology. Whereas in both cases of plasma ferritic nitrocarburizing smooth and fluent friction coefficient graphs were obtained, in the case of gaseous ferritic nitrocarburizing a significant fluctuation of the friction coefficient values during the first 500 s was visible. It could be caused by a warping of the porous part of the white layer. 

The figures and the cross-sectional profiles of the scratches, obtained by the scratch test measurement at three different loads, 15 N, 20 N and 35 N, were utilized for the wear resistance assessment. The observation and measurements showed that significant increases in the surface wear resistance by application of the selected chemical heat treatments were reached. The best results were in the case of 6-hour plasma ferritic nitrocarburizing, where increasing load caused only a minor increase in the scratch dimensions and solely a cohesive failure in the scratch track. Similar results were almost observed in the case of the shortened plasma process, but the load of 35 N caused a greater increase of the track’s dimensions in comparison with other chemical heat treatments. While in the cases of plasma ferritic nitrocarburized surfaces adhesive failures of the white layer at neither load appeared, 15 N was already enough for an occurrence of unexpected delamination in the white layer on the gaseous ferritic nitrocarburized surface. As well as in the case of the friction coefficient measurement, the phenomenon could be associated with an existence of the porous part of the white layer which is brittle and thus prone to be delaminated.

## 5. Conclusions

The information listed above is possible to be summarized into a statement: steel equivalent 1.5752 (i.e., CSN 41 6426) is appropriate for the selected ferritic nitrocarburizing technologies. In cases where deep surface layers are not demanded, plasma ferritic nitrocarburizing seem to be more appropriate for a reason, that reason being that thin and pore-less white layers, providing enhancement in corrosion resistance, can be obtained. Thus, in comparison with frequently utilized carburizing, for an economic reason, the plasma ferritic nitrocarburizing could be a more convenient surface treatment due to the absence of a need for subsequent machining and the shorter exposure time of this technology.

## Figures and Tables

**Table 1:** Parameters of heat treatment.

Parameter	Hardening	Tempering
Temperature (°C)	870	600
Time (min)	20	60
Medium	Water	Water

**Table 2:** Chemical composition of steel equivalent 1.5752 (i.e., CSN 41 6426); instrument: Tasman Q4 Bruker (wt.%).

C	Mn	Si	Cr	Ni	P	S
OES/Bulk						
0.12	0.49	0.33	0.76	3.20	0.020	0.006
Datasheet						
0.10–0.17	0.30–0.60	0.17–0.37	0.60–0.90	2.70–3.20	<0.035	<0.035

**Table 3:** The values of roughness profile parameters of surfaces subjected to the selected chemical heat treatments.

Parameter		Ground Surface	Gaseous Ferritic Nitrocarburizing; 6 h	Plasma Ferritic Nitrocarburizing; 6 h	Plasma Ferritic Nitrocarburizing; 4 h
Ra	(µm)	0.0705	0.2200	0.1020	0.0671
Rq	(µm)	0.1110	0.2770	0.1430	0.1090
Rt	(µm)	1.4300	1.9600	1.6200	1.5300
Rz	(µm)	1.3400	1.7600	1.4600	1.3900
RSm	(mm)	0.0130	0.0207	0.0161	0.0146

Note: measurement parameters were selected as follows: lr = 0.8 mm, ln = 4 mm.

**Table 4:** Values of cross-sectional profile parameters.

Parameter	Load	Heat Treated	Gaseous FNC	Plasma FNC; 6 h	Plasma FNC; 4 h
Width (µm)	15 N	81.67	80.50	62.70	63.80
	20 N	93.50	82.83	70.17	74.77
	35 N	123.00	95.73	83.80	91.60
Depth (µm)	15 N	2.62	1.15	0.64	0.87
	20 N	3.77	1.43	1.01	1.12
	35 N	8.43	2.62	2.11	2.84
Area (µm^2^)	15 N	156.67	58.23	22.43	30.67
	20 N	256.33	82.13	42.77	54.37
	35 N	765.33	186.00	132.67	186.33

## Data Availability

Data are contained within the article.
